# Distinct MRI pattern of “pseudoresponse” in recurrent glioblastoma multiforme treated with regorafenib: Case report and literature review

**DOI:** 10.1002/ccr3.4604

**Published:** 2021-08-21

**Authors:** Lidia Gatto, Enrico Franceschi, Alicia Tosoni, Vincenzo Di Nunno, Ilaria Maggio, Caterina Tonon, Raffaele Lodi, Raffaele Agati, Stefania Bartolini, Alba Ariela Brandes

**Affiliations:** ^1^ Department of Medical Oncology Azienda USL of Bologna Bologna Italy; ^2^ IRCCS Istituto delle Scienze Neurologiche di Bologna Bologna Italy

**Keywords:** antiangiogenic treatment, glioblastoma multiforme, pseudoresponse, RANO criteria, regorafenib, tyrosine kinase inhibitors

## Abstract

Antiangiogenic agents can induce a distinct MRI pattern in glioblastoma, characterized by a decrease in the contrast enhancement on T1‐weighted images and a simultaneous hyperintensity on T2‐weighted or fluid‐attenuated inversion recovery images.

## Graphical abstract

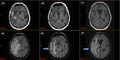

## 1 INTRODUCTION

The radiological assessment for glioblastoma multiforme treated with regorafenib represents a challenge. Although antiangiogenic agents lead to a reduction in tumor contrast enhancement, it does not necessarily reflect a radiological response. We describe an MRI pattern of “pseudoresponse” in a case of glioblastoma treated with regorafenib, characterized by a “T2‐dominant growth pattern.”

Glioblastoma multiforme (GBM) is the most common primary malignant tumor in the central nervous system accounting for more than 48% of all brain tumors.

It has a highly aggressive nature with extremely high morbidity and mortality rate.^1^, ^2^, ^3^, ^4^

The standard treatment for newly diagnosed GBM consists of maximal safe surgical resection followed by concurrent radiotherapy and temozolomide chemotherapy followed by the 6 cycles of chemotherapy alone with temozolomide.

Despite multimodality approach, relapse is inevitable, and the prognosis is poor with a median overall survival of less than 24 months and a 5‐year survival rate of approximately 5%.

In the setting of recurrent disease, the standard of care is less well defined, with little evidence for treatment options prolonging overall survival and a significant proportion of patients no longer eligible for second‐line therapy. Systemic treatment options include nitrosoureas, modestly effective agents, although they were evaluated before routine use of first‐line temozolomide.^5^, ^6^, ^7^, ^8^

The need for additional therapeutic avenues and the evolving insights into the pathophysiology and molecular biology of GBM have allowed the development of new targeted therapeutic approaches: In particular, due to the highly vascularized nature of GBM, a strong interest in targeting “neovascularity” has led to a variety of studies assessing the effectiveness of antiangiogenic agents.^9^, ^10^, ^11^, ^12^, ^13^, ^14^

Bevacizumab, a humanized monoclonal antibody against VEGF, received accelerated approval from the US Food and Drug Administration for treatment of recurrent GBM in the United States, based on the success of two Phase II clinical trials ^9^, ^10^ showing an improvement in progression‐free survival compared with nitrosourea‐based treatment, without, however, proven positive effect on overall survival; as a result, its role in the treatment of recurrent GBM remains unclear.

Nevertheless, antiangiogenic treatments are biologically active in GBM, and other agents have demonstrated favorable antitumor activity.

The REGOMA phase II trial demonstrated the superiority of the antiangiogenic tyrosine kinase inhibitor regorafenib compared to lomustine, in the treatment of recurrent GBM, with significant improvement of the median overall survival from 5.6 to 7.4 months.^15^

The clinical practice with antiangiogenic therapy in GBM has pinpointed the issue of the radiological assessment of disease progression: Since angiogenesis is inhibited, blood‐brain barrier is less disrupted and MRI may detect a decrease in tumor enhancement; however, a large proportion of patients exhibit a diffuse infiltrating non‐enhancing tumor progression after 3–5 months, suggesting that antiangiogenic therapy can be responsible for a more aggressive GBM phenotype, with a pattern of diffuse progression.^16^

These MRI alterations are well known for GBM patients treated with bevacizumab but are poorly described in patients treated with regorafenib, and have required a revision of the response assessment in neuro‐oncology (RANO) criteria, to include the detection of non‐enhancing T2/fluid‐attenuated inversion recovery (FLAIR) lesions as a new criterion for GBM progression.^17^

Herein, we describe a distinct “T2‐FLAIR dominant” MRI pattern of pseudoprogression in a case of recurrent GBM treated with regorafenib, partially resembling a MRI pattern largely described for bevacizumab treatment.

A more accurate, standardized, and reproducible assessment of treatment response seems mandatory, since regorafenib is associated with a spectrum of adverse effects heavily impacting on the quality of life: in the REGOMA trial, in fact, 59% of patients in the regorafenib arm have experienced grade 3–4 adverse events.

## 2 CASE PRESENTATION

A 61 year‐old man was admitted to our hospital at the end of March 2019 with a history of headache lasting for 10 days and sudden onset of left hemiplegia. Brain MRI scans indicated a space‐occupying enhancing hemorrhagic lesion with surrounding edema in the right temporal lobe. Urgent surgical resection of the lesion was performed, and histopathological examination revealed a wild‐type IDH1 GBM. Tumor cells tested positive for *O*‐*6*‐*methylguanine*‐*DNA methyltransferase (MGMT*) status by methylation‐specific polymerase chain reaction (PCR).

A 48‐h postoperative MRI confirmed the presence of residual disease. Adjuvant radiotherapy for a total of 60 Gy in 30 fractions over 6 weeks with concurrent temozolomide (75 mg/m^2^ daily) followed by 6 adjuvant cycles of temozolomide [200 mg/m^2^, days 1–5, every 28 days (q28d)] was given.

**FIGURE 1:**
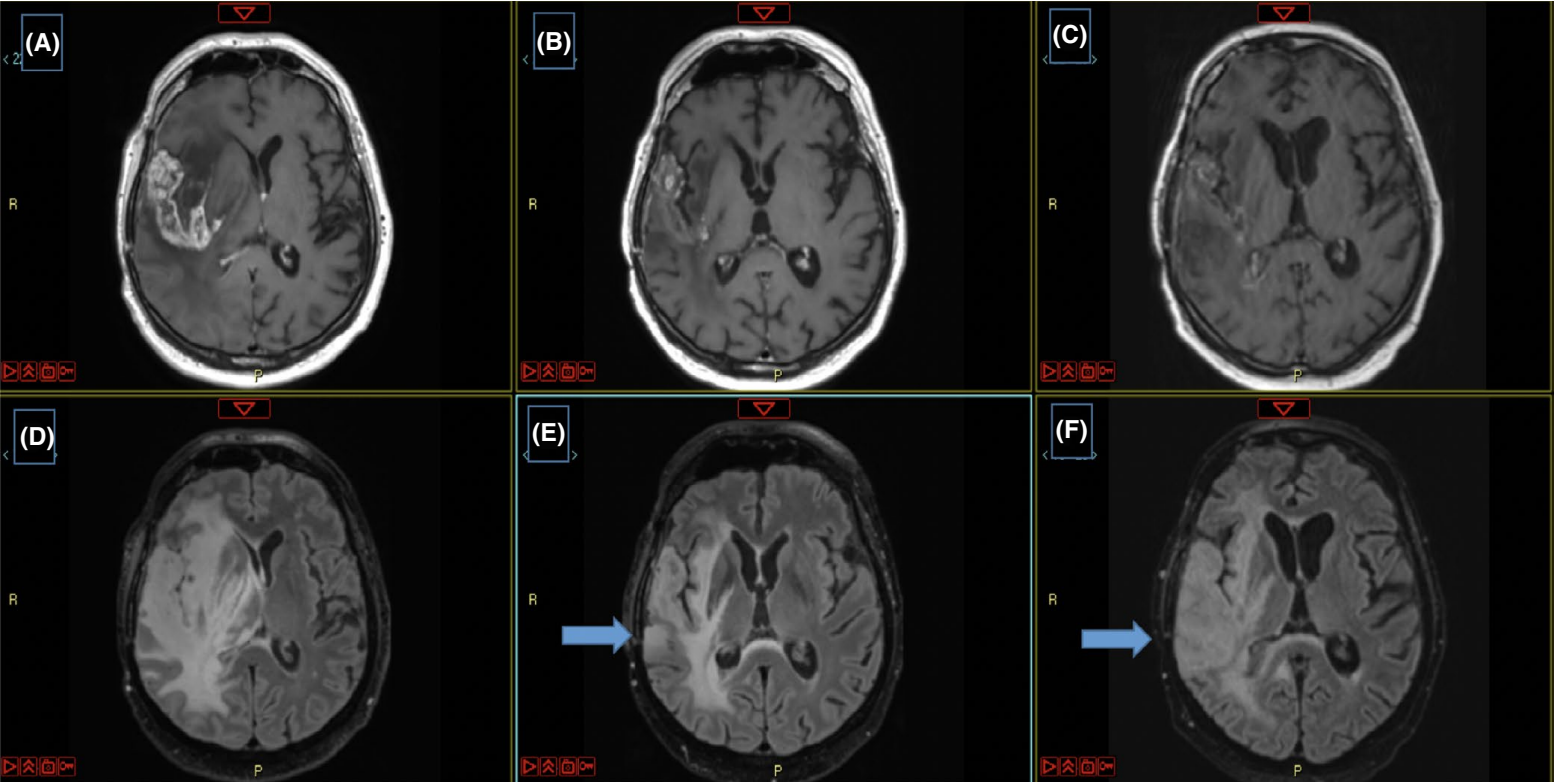
(A) T1 post‐contrast MRI before regorafenib treatment. (B) T1 post‐contrast MRI 3 months after starting regorafenib. (C) T1 post‐contrast MRI 6 months after starting regorafenib. (D) T2/FLAIR MRI before regorafenib treatment. (E) T2/FLAIR MRI 3 months after starting regorafenib. (F) T2/FLAIR MRI 6 months after starting regorafenib

The patient had a follow‐up MRI after 6 cycles of temozolomide, that revealed progression of the cystic mass occupying the entire right temporal lobe with significant increase of the perilesional edema and shift of the midline (Figure 1A and D).

This prompted a new treatment approach consisting of regorafenib, administered at a dosage of 160 mg per day for 21 consecutive days followed by a pause of 7 days. Three months after starting regorafenib, the MRI showed a partial response, with decrease of the contrast enhancement (CE) of the temporal lesion and reduction of the perilesional edema on T1‐weighted images (Figure 1B). However, a shading non‐enhancing area hyperintense in T2‐weighted images was already identifiable in the right posterior temporal region (Figure 1E).

Despite radiological response, the patient showed no clinical and neurological benefit, complaining of headache and imbalance. Nevertheless, given the good radiological response, we decided to continue regorafenib over a course of three additional cycles.

After 6 months of regorafenib treatment, the MRI confirmed the decrease of the enhancing lesion on T1‐weighted images (Figure 1C) but a significant growth of the non‐enhancing area located in the right posterior temporal region, evident in T2‐weighted sequence (Figure 1F).

Clinically, the patient continued to deteriorate, with a progressive worsening of the performance status, becoming bedridden, and in the end was sent for palliative care at home.

## 3 DISCUSSION

The treatment of recurrent GBM is a major challenge of daily neuro‐oncology practice: There is no standard systemic therapy in the second‐line setting, benefits are modest, and the quality of data for all the regimens is poor.^5^, ^6^, ^7^, ^8^, ^18^, ^19^ Therefore, patients are often confronted with an exhausted therapeutic arsenal, but new treatment approaches are emerging.

The angiogenesis is a hallmark of GBM and remains an important therapeutic target in its treatment, especially for recurrent GBM. The monoclonal anti‐VEGF antibody, bevacizumab, widely used in colon cancer ^20^ was a particularly attractive candidate and despite early studies were promising, leading to an accelerated approval by US Food and Drug Administration (FDA) based on phase II data, bevacizumab failed to show a positive effect on overall survival.^9^, ^10^

As a result, bevacizumab is approved in the United States, but not in Europe and its role in the treatment of patients with recurrent GBM remains unclear.

The randomized, open‐label phase II REGOMA trial investigating the multikinase inhibitor regorafenib in recurrent GBM sparked hope for the successful employment of a targeted therapy approach in GBM, significantly improving overall survival (OS) and progression‐free survival (PFS) compared with lomustine.^15^ Regorafenib is a tyrosine and serine‐threonine kinase inhibitor targeting angiogenic (VEGFR), stromal (PDGFR, FGFR), and oncogenic (KIT, RAF, RET) tyrosine kinase receptors.^21^

Antiangiogenic agents produce a stabilizing effect on the hematoencephalic barrier resulting in a decrease of the vasogenic edema and of the mass effect, with slight improvement of the patients’ symptoms and quality of life, but this does not always correspond to an effective regression of the disease.

It is demonstrated that, if on the one hand, an initial reduction in the enhancement of the tumor mass is observed, on the other, when blocking angiogenesis with bevacizumab, GBM’s growth pattern changes and become more infiltrative.^22^, ^23^, ^24^

This growth pattern change results in distinct MRI alterations consisting in “T2‐dominant growth pattern,” that is a decrease of CE on T1‐weighted MRI despite the appearance of a progressive, infiltrative non‐enhancing growth pattern on T2/FLAIR images.

“Pseudoresponse” was coined to describe this phenomenon, observed in 20%–60% of patients treated with bevacizumab, characterized by a decrease in the tumor enhancement according to Macdonald's criteria, without a true antitumor effect.^25^, ^26^ It was considered as one of the most proper explanations for the discrepancy between the substantial objective response on MRI and poor overall survival rates. Due to this phenomenon, the conventional Macdonald criteria, based on the assessment of enhancing lesions, were found to be inadequate and were replaced by the Response Assessment in Neuro‐Oncology (RANO) criteria. They take into account the issue of pseudoresponse with antiangiogenic agents and include the assessment of T2/FLAIR abnormalities as an additional marker for tumor progression. RANO criteria do not establish a cutoff for the detection in T2/FLAIR of progressive disease for non‐enhancing lesions; however, a ≥25% increase of non‐enhancing lesions in T2/FLAIR may be likely considered the cutoff of progressive disease.^27^

Nowosielski et al. ^16^ performed a retrospective study analyzing the type of radiologic progression in patients treated with bevacizumab, and categorized four progression types:

1. T1 flare‐up progression, that is initial decrease in and subsequent flare‐up of the CE at progression
2. T2‐diffuse progression without new or only speckled CE
3. T2 circumscribed progression without new CE
4. primary nonresponders, that is, no decrease in CE or development of new lesions at first follow‐up imaging.

They observed that patients presenting a T2‐diffuse pattern of disease progression showed longer survival, while primary nonresponders had poorer survival.

It has been shown that adding the T2/FLAIR criteria to the response assessment in recurrent GBM treated with bevacizumab reduces the response rates by approximately 5% and may lead to earlier detection of progression.^28^

In this paper, we describe a case of pseudoresponse to regorafenib partially resembling MRI patterns described for bevacizumab treatment.

In our case, a T2‐dominant growth pattern has been observed despite initial decrease in tumor‐enhancing T1‐weighted images.

Practically, since CE decreased in T1‐weighted images at first follow‐up MRI, we detected an infiltrating non‐enhancing recurrence, accompanied by progressive clinical deterioration, which preceded by about three months the detection of radiological disease progression established by the classic Macdonald and Response Evaluation Criteria in Solid Tumors (RECIST).

While the issue of pseudoresponse is well known for bevacizumab treatment, for regorafenib it is poorly described in literature.^28^

Zeiner et al. were the first to categorize the disease progression in GBM patients treated with regorafenib into two distinct MRI patterns: the *classic progressive disease pattern*, defined by a 25% or more increase in enhancing lesions on T1 post‐contrast imaging, or the *T2*‐*dominant growth pattern* characterized by an overall decrease in CE on T1 post‐contrast imaging and, simultaneously, an increase in non‐enhancing tumor on T2 imaging. Patients with a *T2*‐*dominant growth pattern* showed a significantly better survival (median survival of 27 weeks from initiation of regorafenib treatment) in contrast to patients with classic disease progression that had a median survival of 10 weeks.^28^

Despite current research on antiangiogenic treatment, the observation that GBM may progress also without the process of neoangiogenesis and the understanding of biological mechanisms of tumor escape like vessel “co‐option,” a mechanism in which tumor cells obtain a blood supply by migrating along preexisting vessels,^22^ explain how the benefits of these drugs are often transient, and tumors rapidly progress.

Improving radiological assessment in recurrent GBM treated with regorafenib remains a challenge: MRI interpretation is not easy, but, new imaging techniques are emerging to overcome this limitation.

The PET/RANO group ^29^ suggested the use of amino acid positron emission tomography (PET) using O‐(2‐[^18^F]‐fluoroethyl)‐L‐tyrosine (FET) as a valuable tool for response assessment in GBM, especially during antiangiogenic treatment. In a small case series, FET‐PET was able to detect both pseudoresponse and pseudoprogression and allowed earlier diagnosis of tumor progression although MRI findings were unchanged during follow‐up.^30^

One of the most substantial weakness of RANO criteria is the poor accuracy in discriminating between non‐enhancing progressive tumor and other causes of hyperintensity on T2‐FLAIR such as vasogenic edema, microvascular ischemic events, and leukoencephalopathies. Advanced MRI sequences that allow the functional assessment of tumors can overcome this limitation, such as diffusion‐weighted imaging (DWI), MR spectroscopy, and perfusion‐weighted imaging.^19^ DWI may be considered as one of the subsidiary tools for the detection of the invasive non‐enhancing tumor in patients underneath antiangiogenic therapy. Possibly, variations in N‐acetylaspartate, choline peaks on MR spectroscopy can be used as a reliable imaging biomarker for discriminating between response and pseudoresponse and between vasogenic edema and non‐enhancing tumor.

## 4 CONCLUSION

GBM remains the most aggressive malignancy of the central nervous system despite long‐standing efforts to improve treatment options and diagnostic tools.

Regorafenib and antiangiogenic agents created an open debate about the imaging assessment of GBM response in both clinical trials and clinical practice.

Measuring and interpreting changes in T2 and T2/FLAIR MRI pattern to identify the presence of non‐enhancing disease progression in patients with recurrent GBM under regorafenib treatment are challenging in order to avoid overestimation of radiological response and PFS.

This is even more important when considering the toxicity profile of regorafenib, which is often burdened with major side effects that impact on quality of life.

The development of advanced imaging tools such as amino acid PET, MR diffusion, perfusion, and spectroscopy to evaluate the therapeutic effect of novel antiangiogenic agents and the standardization of neuro‐radiological response patterns are challenging for future prospective research.

## CONFLICT OF INTEREST

All authors declare no conflict of interest.

## AUTHOR CONTRIBUTIONS

L.G. and E.F. involved in conceptualization, and writing original draft preparation. L.G., E.F., and A.A.B. involved in writing review and editing. All authors involved in supervision, have read and agreed to the published version of the manuscript.

## ETHICAL STATEMENT

The patient has signed a informed consent.

## Data Availability

The data that support the findings of this study are available from the corresponding author upon reasonable request.
